# Accommodative Stimulus-Response Curve with Emoji Symbols

**DOI:** 10.1155/2017/4165706

**Published:** 2017-09-10

**Authors:** Robert Montés-Micó, José J. Esteve Taboada, Paula Bernal-Molina, Teresa Ferrer-Blasco

**Affiliations:** ^1^Department of Optics and Optometry and Visual Sciences, University of Valencia, 46100 Burjassot, Spain; ^2^Interuniversity Laboratory for Research in Vision and Optometry, Mixed Group UVEG-UMU, Valencia, Spain

## Abstract

**Purpose:**

To evaluate the static measurement of the accommodative stimulus-response curve with emoji symbols.

**Methods:**

The accommodative stimulus-response curve was measured in 18 subjects using a Hartmann-Shack sensor to obtain the objective accommodative response from the Zernike defocus term. Measurements were acquired at different accommodative demands, from 0 to 3 D with a step of 0.5 D. Detailed and nondetailed emoji targets were used with two different sizes, corresponding to the two most common visual angles used in smartphones.

**Results:**

A regression analysis was performed to fit the mean results obtained for each target. The determination coefficient was *R*^2^ ≥ 0.988 for all targets. For the detailed targets, the slopes for the averaged stimulus-response curve were 0.65 and 0.66 for the bigger and smaller sizes, respectively. For the nondetailed targets, the slopes were 0.60 and 0.58 for the bigger and smaller sizes, respectively. *p* values for these slopes were statistically significant for the two types of targets (*p* < 0.01).

**Conclusions:**

Our results reveal that the replacement of a word or several words by detailed or nondetailed emoji symbols seems not to provoke a different accommodative response in normal subjects and under standard viewing conditions in the use of smartphones.

## 1. Introduction

Over the last few years, there has been a significant increase in the use of Internet-based instruments such as tablets and/or smartphones. Around two billion phones are estimated as currently in use worldwide, and this number is expected to double by 2020 [1]. The number of hours of use in front of a tablet or smartphone screen is increasing, both in adults and children. In addition, the reading distance may vary since smaller screen size implies that the user tends to get closer for its use. Bababekova et al. [2] reported that the mean viewing distance for reading text messages on a smartphone is 4 cm closer than for Internet viewing, in both cases closer than that usually adopted for other computer devices. Also, there is a study that shows that the viewing distance of a smartphone may vary depending on the time spent reading, being closer after 60 minutes [3].

Smartphones can be used for different visual tasks such as reading the Internet, viewing videos, and writing and reading messages in social networks or messaging applications. These activities are changing quickly, and it may be now found that some written text is replaced by emojis. An emoji is defined as a pictograph (graphic symbol) that represents not only a facial expression but also concepts and ideas [4] and is frequently used to replace some words in messages. To replace a word or several words by an emoji may provoke a different accommodative response (AR) depending on the details included in the emoji symbol. The assessment of the AR by means of the accommodative stimulus-response curve may provide information regarding some relevant clinical aspects. The analysis of this curve is important for the assessment of the relationship between accommodation and myopia or amblyopia development [5–8].

Therefore, the purpose of the present study is to evaluate the accommodative stimulus-response curve using a wavefront-sensing optical system in order to assess the effect of detailed and nondetailed emoji symbols used in messaging applications frequently found in smartphones.

## 2. Methods

### 2.1. Subjects

Eighteen young adult subjects with a mean age of 28.6 ± 8.2 years were recruited for this experiment. The mean spherical equivalent refractive error was −0.16 ± 1.30 diopters (D). Astigmatism was limited to ≤1.00 D and anisometropia <2.00 D. All subjects had a best-corrected visual acuity of 20/20 or better, showed no ocular pathology, no previous conducted ocular surgery, and normal clinical amplitudes of accommodation for their ages. The study followed the Declaration of Helsinki and was approved by the Ethics Committee of the Institution. The subjects were verbally informed about the details and possible consequences of the study, and a signed formal consent was obtained from each subject.

### 2.2. Experimental System

**Figure 1:**
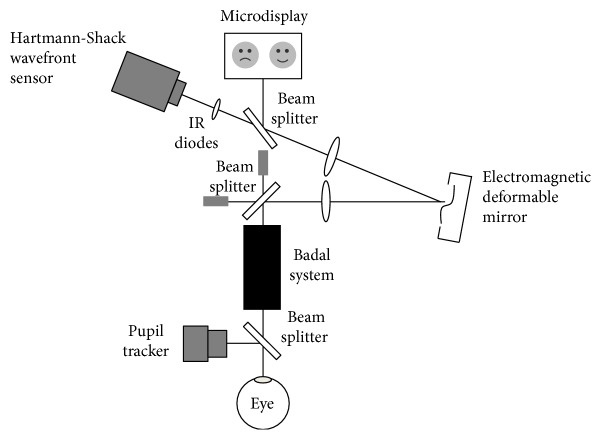
Schematic diagram of the wavefront-sensing optical system.

A wavefront-sensing optical system was used to carry out the measurements.  Figure 1 shows a detailed description of the experimental setup used. The system is composed of a Hartmann-Shack wavefront sensor (Haso 32, Imagine Eyes, France) and a 52-actuator deformable mirror (Mirao 52, Imagine Eyes, France) that was used to compensate for the internal aberrations of the optical system [9]. The wavefront sensor employs a square array of 1024 microlenses and a near-infrared light source with a wavelength of 850 nm. An internal microdisplay is used to project the target, while the Badal system is employed to change the accommodation demand. A precise alignment of the subject's pupil is required, which was controlled with an additional camera. Head movements were reduced employing a chin and forehead rest. The subject's right eye viewed the target and the left eye was patched. All measurements were taken using the analysis and simulation software library and software development kits provided by the manufacturer (Imagine Eyes, France).

### 2.3. Experimental Procedure

**Figure 2:**
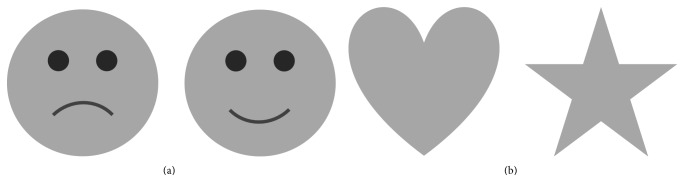
Detailed (a) and nondetailed (b) emoji symbols used for the experiment.

Measurements were acquired at different accommodative demands (AD), from 0 to 3 D with a step of 0.5 D. Detailed (happy and sad smileys) and nondetailed (heart and star) emoji targets, so classified depending on the level of details included in the emoji symbols, were used with two different sizes (21 and 30 arc min), corresponding to the two most common visual angles used in smartphones at a standard distance of 30 cm. These two sizes were selected according to the most used configuration for viewing messages in end-to-end instant messaging applications. Detailed emojis are supposed to require higher visual acuity than nondetailed ones (see Figure 2 for a description of both emoji targets). The accommodative stimulus-response curve was measured under four different conditions, combining both the type and the size of emojis. Subjects were also allowed to rest between trials.

### 2.4. Data Analysis

Wavefront data were exported as Zernike coefficients up to the 6th order. In order to identify the AR of the eyes to the accommodation stimuli only, Zernike defocus was used. AR was determined in diopters by using the equation that follows:

(1) $$\begin{array}{c} AR=AD-\frac{C_{2}^{0}4\sqrt{3}}{r^{2}}, \end{array}$$

where *C*_2_^0^ is the second-order Zernike coefficient for defocus, expressed in *μ*m, and *r* is the pupil radius, expressed in mm [10].

Mean data for each of the different conditions were fitted into linear models. For each regression analysis, the following values were recorded: intercept *n*, slope *m*, determination coefficient *R*^2^, and *p* values. Besides these values, the accommodative error index *I* was obtained for each condition [11]. This metric is defined as the mean of the response error magnitude divided by the squared correlation coefficient. The accommodative error index is defined therefore as

(2) $$\begin{array}{c} I=\frac{\left|\left(1-m\right)\left(\left(x_{1}+x_{2}\right)/2\right)-n\right|}{R^{2}}, \end{array}$$

where *x*_1_ and *x*_2_ correspond to the stimulus levels defining the range over which the regression fit applies. Slope values themselves are not valid to characterize a stimulus-response curve, since a curve with *m* = 1 does not necessarily coincide with the ideal line and important lags or leads may still be present, and therefore, the accommodative error index was introduced to consider both the extent to which responses deviate from ideal and the goodness of fit of data points to the regression line. The accommodative error index *I* value increases with the numerator value, that is, when the discrepancy between the regression line fitted to the measured accommodative responses and the ideal line increases (measured by the normalized area between both lines). The value of *I* also increases as the denominator decreases, that is, when the degree of correlation between stimulus and response is not high.

An additional ANCOVA analysis using MATLAB 2015b (The MathWorks Inc., Natick, MA, USA) was also carried out in order to determine whether the slopes of the four different conditions were significantly different. A *p* value lower than 0.05 was considered as statistically significant.

## 3. Results

**Figure 3:**
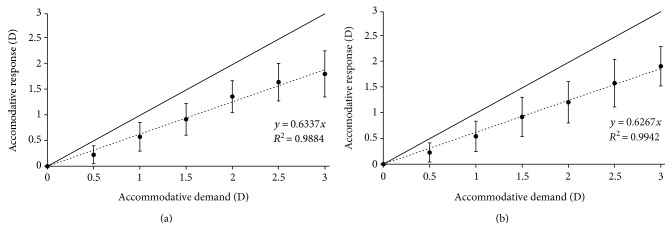
Mean accommodative response obtained with the detailed (happy and sad smileys) emoji targets for 30 (a) and 21 (b) arc min. Each data point represents the mean ± standard deviation (SD) at each accommodative demand. The solid line represents the theoretical accommodative response while the dotted line represents the fitted linear model.

Figure 3 shows the mean AR obtained for all the subjects for each AD, starting from 0 D and ending at 3 D of AD with a 0.5 D step with the detailed (happy and sad smileys) emoji targets for 30 (top) and 21 (bottom) arc min. The slopes for the averaged stimulus-response curve were 0.634 and 0.627 for the bigger and smaller sizes, respectively. The *p* values for these slopes were statistically significant for the two sizes (*p* < 0.01). The determination coefficient in both cases was *R*^2^ ≥ 0.988.

**Figure 4:**
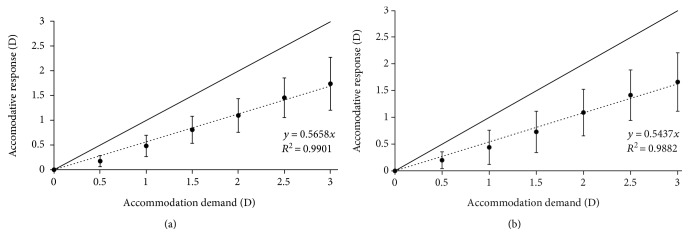
Mean accommodative response obtained with the nondetailed (heart and star) emoji targets for 30 (a) and 21 (b) arc min. Each data point represents the mean ± standard deviation (SD) at each accommodative demand. The solid line represents the theoretical accommodative response while the dotted line represents the fitted linear model.

Figure 4 shows the averaged AR obtained with the nondetailed (heart and star) emoji targets for 30 (top) and 21 (bottom) arc min. The slopes for the averaged stimulus-response curve were 0.566 and 0.544 for the bigger and smaller sizes, respectively. The *p* values for these slopes were statistically significant for the two sizes (*p* < 0.01). The determination coefficient in both cases was *R*^2^ ≥ 0.988.

The solid line in both figures shows the theoretical ideal response of the accommodation process (i.e., equal AR for each AD). In this case, there was a difference towards the same direction between all AR and the theoretical line, showing accommodative lag for all subjects and AD. The accommodative error index *I* values for the different conditions assessed were the following: 0.56 and 0.56 for the detailed emoji targets for 30 and 21 arc min, respectively and 0.66 and 0.69 for the nondetailed emoji targets for 30 and 21 arc min, respectively.

An ANCOVA statistical analysis was conducted to analyze if the measurements obtained for the four different conditions were statistically different or not. This analysis revealed that the slopes of the AR for the four conditions were not significantly different from each other (*p* = 0.06). Despite no statistically significant differences were found in the slope values, the outcomes for the detailed emoji targets were slightly larger than the ones obtained for the nondetailed emoji targets.

## 4. Discussion

As we have introduced, there is a general trend worldwide to increase the number of hours using handheld devices such as smartphones or tablets. The use of these devices, specifically by children and young individuals, may produce a change in their AR. It is interesting to note, for example, that smartphone gaming has risen dramatically in recent years [12]. Specifically, smartphone gaming and frequent use patterns are associated with smartphone addiction [13]. For example, Haug et al. [14] indicated that smartphone addiction occurred in 16.9% of a sample of 1519 students from Switzerland. Then, the excessive use of a smartphone can be described as a type of behavioural addiction that changes several aspects of information reading and communication. The addiction that is actually reported in the literature produces inevitably an increasing number of hours of use that may affect the AR.

The use of instant messaging applications frequently found in smartphones is one of the main factors that contributes in increasing the number of hours of use. An emoji, defined as a pictograph that describes concepts, ideas, and emotions, is one of the communication elements most used in these applications, to such an extent that frequently the communication between individuals only considers emoji symbols. Then, when text is replaced by an emoji symbol, this may also affect the AR and should be evaluated. In addition, it becomes important for the user of these applications to pay detailed attention to the emoji symbol used, since, for instance, emojis representing different emotions or feelings differ only in small details (see, for example, happy and sad smileys in Figure 2(a)).

Therefore, the aim of the present study focuses on the analysis of the accommodative stimulus-response curve using a wavefront-sensing optical system in order to properly analyze the effect of emoji symbols on the accommodative system of the human eye. Due to the importance of the details in some emojis to properly communicate the message, we have considered the analysis with two different groups of emojis (detailed and nondetailed) requiring different visual demanding tasks.

Our results revealed that under all the conditions of the experiments that were carried out there was an accommodative lag for all subjects evaluated. Mean lag values for all the experimental conditions increased for higher vergences, ranging from 0.3 (at 0.5 D) to 1.3 D (at 3 D). These results agree with previous literature showing that accommodative lag increases with AD [7, 15–17]. We have to note that there have been reported differences in the accommodative stimulus-response curves depending on the measurement method used [7, 17]. Recently, Chen et al. [17] measured monocularly this curve using three methods: dynamic and static measurements using a motorised Badal system and the minus lens technique. They concluded that the results are method-dependent, and that using dynamic measurements, accommodative behaviour varies with the speed of dioptric change of the stimulus. In our experiment, we have used a Hartmann-Shack wavefront sensor with a Badal lens, and the accommodation measurement was based on the Zernike defocus term (see (1)).

Figures 3() and 4() show the data for the detailed and nondetailed emoji symbols used. Specifically, Figure 3() shows the happy and sad smiley emoji targets for 30 and 21 arc min. The slopes for both types of symbols were higher than 0.6 and the determination coefficient *R*^2^ ≥ 0.988, being statistically significant for the two sizes evaluated (*p* < 0.01). Similar outcomes were found for the heart and star emoji targets (see Figure 4()). The slopes were higher than 0.5 and the determination coefficient *R*^2^ ≥ 0.988, being statistically significant for the two sizes evaluated (*p* < 0.01). The ANCOVA analysis revealed that there were no statistically significant differences between the results obtained for the slopes in the four experimental conditions (*p* = 0.06), although it is interesting to point out that the slopes for the detailed emoji targets (Figure 3()) were slightly larger than the ones obtained for the nondetailed emoji targets (Figure 4()). This response may be due to the fact that detailed targets may require higher AR to reduce the accommodative lag and increase the quality of the retinal image [16]. In relation to the emoji sizes, our results revealed that there were no differences between the slopes (see Figures 3() and 4()). Some instant messaging applications that are commonly used worldwide increase the size of the emoji when it is displayed alone, without text. Despite the fact that there are no differences in the AR, this different size may allow for a better visualization increasing the reading speed or message comprehension, for example. In relation to the accommodative error index, the results obtained revealed and confirmed that the detailed emoji targets require larger AR to reduce the accommodative lag. Note, for example, that the accommodative error index for nondetailed targets is about 25% higher than the values obtained for the detailed targets.

One of the limitations in our study is that we have used some specific emojis. However, there are a lot of them and new ones appear every day. Besides, the use of a particular emoji symbol changes frequently by users. In this regard, for example, the website Emojitracker (http://emojitracker.com/), that monitors the use of emojis on Twitter in real time, shows how the frequency of use for a particular emoji changes with time. Future studies should be done with more emoji types.

In conclusion, we have measured the accommodative stimulus-response curve under different conditions combining both the type and the size of emoji symbols. Our results reveal that the replacement of a word or several words by detailed or nondetailed emoji symbols seems not to provoke a different AR in normal subjects and under standard viewing conditions in the use of smartphones. However, further research should be carried out in order to evaluate the use of other emoji symbols that appear continuously.
